# Gp130 is expressed in pancreatic cancer and can be targeted by the small inhibitor molecule SC144

**DOI:** 10.1007/s00432-022-04518-9

**Published:** 2022-12-10

**Authors:** Ioannis Pozios, Nina A. Hering, Emily Guenzler, Marco Arndt, Sefer Elezkurtaj, Thomas Knösel, Christiane J. Bruns, Georgios A. Margonis, Katharina Beyer, Hendrik Seeliger

**Affiliations:** 1 Department of General and Visceral Surgery, Charité—Universitätsmedizin Berlin, corporate member of Freie Universität Berlin and Humboldt-Universität zu Berlin, Hindenburgdamm 30, 12203 Berlin, Germany; 2 Institute of Pathology, Charité—Universitätsmedizin Berlin, corporate member of Freie Universität Berlin and Humboldt-Universität zu Berlin, 10117 Berlin, Germany; 3 Institute of Pathology, University Hospital, Ludwig-Maximilians-University Munich, 81377 Munich, Germany; 4 Department of General, Visceral, Tumor and Transplantation Surgery, University Hospital of Cologne, University of Cologne, Cologne, Germany; 5 Department of Surgery, Memorial Sloan Kettering Cancer Center, New York, NY USA; 6 IU Health University, 55116 Mainz, Germany

**Keywords:** SC144, Pancreatic cancer, Interleukin 6, Oncostatin M, gp130, STAT3

## Abstract

**Purpose:**

Interleukin 6 (IL-6), Oncostatin M (OSM), and downstream effector STAT3 are pro-tumorigenic agents in pancreatic ductal adenocarcinoma (PDAC). Glycoprotein 130 (gp130) is a compound of the IL-6 and OSM receptor complex that triggers STAT3 signaling. SC144 is a small molecule gp130 inhibitor with anticancer activity. This study examines the gp130 expression in human PDAC specimens and the in vitro effects of SC144 in PDAC cell lines.

**Methods:**

Tissue micro-arrays were constructed from 175 resected human PDAC. The gp130 expression in tumor epithelium and stroma was determined by immunohistochemistry, and survival analysis was performed. Growth inhibition by SC144 was assessed in vitro using BrdU and MTT assays. Western blotting was performed to evaluate the SC144 effect on IL-6 and OSM signaling.

**Results:**

Gp130 was expressed in the epithelium of 78.8% and the stroma of 9.4% of the tumor samples. The median overall survival for patients with or without epithelial gp130 expression was 16.7 months and 15.9 months, respectively (*p* = 0.830). Patients with no stromal gp130 expression showed poorer survival than patients with stromal gp130 expression (median 16.2 and 22.9 months, respectively), but this difference did not reach significance (*p* = 0.144). SC144 inhibited cell proliferation and viability and suppressed IL-6- and OSM-stimulated STAT3^Y705^ phosphorylation in PDAC cells.

**Conclusion:**

Gp130 is expressed in the epithelium of most human PDAC, but stromal expression is rare. The small molecule gp130 inhibitor SC144 potently inhibits PDAC progression in vitro and may abrogate IL-6 or OSM/gp130/STAT3 signaling. These results suggest gp130 as a novel drug target for pancreatic cancer therapy.

## Background

Malignancies of the pancreas account for about three percent of all cancers but remain nowadays the fourth most common cause of cancer-related death in both sexes in the western world (Torre et al. 2015; Siegel et al. 2022). By 2030, total deaths from pancreatic cancer are supposed to increase dramatically and become the second leading cause of cancer-related deaths after lung cancer (Rahib et al. 2014). Despite the continuous progress in the field of chemotherapy, the medical treatment’s impact on the natural history of the disease remains pure (Denley et al. 2013). Thus, novel therapeutic agents are urgently required to improve the prognosis of pancreatic cancer.

In pancreatic ductal adenocarcinoma (PDAC), key signaling pathways are dysregulated, contributing to pancreatic tumorigenesis. Recent studies have shown that interleukin 6 (IL-6) and the major downstream effector signal transducer and activator of transcription 3 (STAT3) are pro-tumorigenic agents in a variety of human cancers, including PDAC (Taher et al. 2018). High serum IL-6 levels have been proposed as a negative prognostic marker in patients with PDAC (Lesina et al. 2014) and correlated with poor survival, weight loss, and cachexia (Bellone et al. 2005; Falconer et al. 1994; Okada et al. 1998). IL-6 is elevated not only in serum but also in tumor tissues isolated from patients with pancreatic cancer.

IL-6 is a pleiotropic cytokine with biological effects on various cells regulating many cellular functions, including cell proliferation, cell differentiation, immune defense mechanisms, and hematopoiesis (Lesina et al. 2014). IL-6 acts either by affecting the tumor cells directly or modulating the tumor microenvironment. A study in KRAS-mutated mice demonstrated the crucial role of IL-6 in PDAC, showing that IL-6 presence and STAT3 activation are necessary for the early PanIN lesions to be developed to PDAC (Lesina et al. 2011). STAT3 is a transcription factor, and its gene is an oncogene expressed in several human cancers, including pancreatic, having a well-established role in tumorigenesis (Corcoran et al. 2011; Yu et al. 2009).

Our previous studies showed a high expression of IL-6/STAT3 pathway proteins in human PDAC specimens (Pozios et al. 2018) and promising results for tumor growth inhibition by blocking this signaling (Pozios et al. 2020). IL-6 mediates part of its functions through the IL-6-receptor complex (IL-6R). Glycoprotein 130 (gp130), as a compound of the IL-6R complex, plays a crucial role in this signaling cascade and is expressed in almost all organs playing a fundamental role in cell survival and growth (Xu and Neamati 2013). Interestingly, gp130 is a common receptor part of the IL-6 family cytokines, as IL-6 is only one of the eleven known cytokines of the IL-6 family, activating the same signaling cascade with similar downstream effects. One of these IL-6 family cytokines is Oncostatin M (OSM), which is overexpressed in the sera of nontreated PDAC patients compared with the healthy volunteers (Torres et al. 2014). OSM is associated with poor prognosis in PDAC and contributes to the epithelial–mesenchymal transition of PDAC cells (Duijneveldt et al. 2020).

The above data indicate IL-6/gp130/STAT3 signaling as a possible target in chemotherapy for pancreatic cancer. A novel small molecule gp130 inhibitor named SC144 was recently discovered with a broad-spectrum anticancer activity without significant toxicity to normal tissues (Oshima et al. 2009; Xu et al. 2013). Although SC144 was shown to act promising in models of ovarian, colon, or breast cancer (Oshima et al. 2009; Xu et al. 2013), its effectivity in PDAC was not studied yet.

The present study aims to analyze the expression of gp-130 on pancreatic tumors and its role in disease progression and survival. Furthermore, we investigate if gp-130 can be targeted by the novel inhibitor SC144 in pancreatic cancer cell lines.

## Methods

### Patients

In total, 211 patients who underwent surgical therapy for PDAC at the Department of Surgery at the Hospital of the Ludwig-Maximilians-University of Munich between 2003 and 2010 were considered for this study. Exclusion criteria were perioperative mortality (patients dying within 30 days after curative resection), macroscopic residual disease after resection, and periampullary tumors other than PDAC, (e.g., ampullary, distal cholangiocarcinomas, and duodenal adenocarcinomas). Finally, 36 patients were excluded, and 175 patients were included in the analysis. Data on clinicopathological parameters and follow-up information were extracted from the local tumor registry and clinical records. The study was approved by the Ethics Committee of the Hospital of the Ludwig-Maximilians-University of Munich.

### Tissue micro-arrays

Archival tumor specimens (paraffin tissues) from the Institute of Pathology of the Ludwig-Maximilians-University of Munich were analyzed, and tissue micro-arrays (TMAs) were constructed according to standard procedures as previously described (Knösel et al. 2005, 2006). Two TMAs containing 422 samples from 211 patients were constructed. The PDAC TMA was assembled using 0.6-mm punch biopsies from all 211 samples. In total, 422 specimens of pancreatic tissue, including normal mucosa, were evaluated.

### Immunohistochemistry

Immunohistological staining of TMAs was performed according to standard procedures. Briefly, the TMA slides were pretreated and then incubated with anti-gp130 antibody (Cell Signaling Technology, Frankfurt am Main, Germany), followed by antibody detection via biotinylated anti-mouse secondary antibody and a biotin–streptavidin amplified detection system (Biogenex, San Ramon, CA, USA). Staining was visualized using a Fastred chromogen system (DAKO, Hamburg, Germany). The TMA slides were evaluated by a person blinded for the clinical data. The staining was scored semiquantitatively by a four-tier scale (0, negative; 1, weak; 2, moderate; 3, strongly positive) according to standard procedures(Knösel et al. 2006). This scoring system was also reduced to a two-tier system (0, negative; 1–3, positive) for the statistical analysis of protein epithelial and stromal expression and its correlation with clinicopathological parameters, including survival.

### Cell lines and reagents

AsPC-1 cells were purchased from the American Type Cell Culture Collection (Virginia, USA). L3.6pl is a secondary, highly metastatic human PDAC cell line of an orthotopic mouse xenograft model (Bruns et al. 1999). Cell lines were maintained in Dulbecco’s Minimal Essential Medium (Invitrogen GmbH, Karlsruhe, Germany), supplemented with 10% fetal bovine serum and 1% penicillin–streptomycin. All cells were incubated in a humidified atmosphere of 5% CO_2_ at 37 °C. Cells were routinely checked for mycoplasma contamination using PlasmoTest (InvivoGen).

SC144 (a quinoxalinhydrazide derivative) was purchased from Sigma-Aldrich (Schnelldorf, Germany) and was solved in 0.1% dimethyl sulfoxide (DMSO). IL-6 was purchased from Invitrogen GmbH (Karlsruhe, Germany) and was dissolved in acetic acid. OSM was obtained from Cell Signaling Technology (Frankfurt am Main, Germany), and dissolved in phosphate-buffered saline. Anti-phosphorylated-STAT3 (Y705), anti-STAT3, and anti-gp130 antibodies were purchased from Cell Signaling Technology (Frankfurt am Main, Germany). Anti-β-actin was purchased from Sigma–Aldrich (Taufkirchen, Germany).

### Target identification using DARTS assay

Drug Affinity Responsive Target Stability (DARTS) assay was performed to identify and assess the protein–ligand interaction between SC144 and gp130. DARTS assay identifies potential protein targets for small molecules via the protease protection from pronase as described previously (Lomenick et al. 2009. 2011). Briefly, L3.6pl cells were lysed using M-PER (Thermo Fisher) supplemented with protease and phosphatase inhibitors. The cell lysate, containing 3 μg/μL total proteins, was treated with SC144 or with solvent alone at room temperature for one hour, followed by proteolysis for 5 min at room temperature with pronase (1:5000 ratio; Roche Applied Science, Penzberg, Germany) as described previously (Lomenick et al. 2011). Proteolysis was stopped by adding SDS loading buffer and heating to 95 °C for five minutes. Afterward, the samples were analyzed by Western blotting as described below.

### Western blot

L3.6pl cells were treated with different concentrations of SC144 or solvent in a serum-free medium. Phosphorylation was stimulated by IL-6 (100 ng/ml) or OSM (50 ng/ml) 3 h after the addition of SC144 (1, 2, 3, and 5 µM). After six or 24 h of incubation, cells were washed with ice-cold phosphate-buffered saline and lysed in radioimmunoprecipitation assay lysis buffer containing phosphatase and protease inhibitors. Protein concentration was determined by Quantipro BCA (bicinchoninic acid) protein assay kit (Sigma–Aldrich, Taufkirchen, Germany) according to the manufacturer’s instructions. The proteins were fractionated by SDS polyacrylamide gel electrophoresis and electrotransferred to polyvinylidene difluoride membranes (Perkin Elmer, Boston, USA). Membranes were blocked (Tris-buffered Saline with Tween 80, 5% Bovine Serum Albumin, and 0.02% sodium azide) and incubated with specific primary antibodies (1:1000) overnight at 4 °C. Then, they were subsequently probed with horseradish peroxidase-conjugated secondary antibody. Signals were visualized by luminescence imaging (Peqlab Biotechnologie GmbH, Erlangen, Germany) using an enhanced chemiluminescent substrate system (SuperSignal West Pico PLUS, Thermo Fisher, Ulm, Germany).

### Measurement of cell proliferation and viability

AsPC-1 or L3.6pl cells were seeded in 96-well plates (5000 cells/well) and were allowed to grow overnight. Then cells were incubated with increasing concentrations of SC144 in a serum-free medium at 37 °C for 48 h. The effect of SC144 on cell proliferation was measured using the 5-Bromo-2′-deoxyuridine (BrdU) incorporation assay, according to the manufacturer’s instructions (Roche Applied Science, Penzberg, Germany).

Cytotoxicity was assessed by a MTT [3-(4,5-dimethylthiazol-2-yl)-2,5-diphenyltetrazolium bromide] assay (Sigma-Aldrich, Missouri, USA) as previously described (Carmichael et al. 1987). MTT reagent (at a final concentration of 0.5 mg/ml) was added to each well, and cells were incubated for four hours at 37 °C. After removing the medium, 10% sodium dodecyl sulfate (SDS) was added, and the absorbance was read at 550 nm. All assays were conducted in sextuplicate.

### Statistical analysis

Data were analyzed with SPSS software, version 20.0 (IBM Corp., Armonk, NY, USA). Statistical analysis and *p* value determinations were carried out by *t* test with a confidence interval of 95% for the determination of significant differences between treatment groups. ANOVAs and the Bonferroni-corrected post hoc tests were conducted for multiple comparisons. Chi-square or Fisher’s exact tests were used for the categorical variables to compare independent groups. Fisher’s exact test was used to analyze categorical data when the sample size was small. *p* values lower than 0.05 were considered statistically significant. Kaplan–Meier curves were performed for each investigated parameter. Survival curves were compared and assessed using the log-rank test.

## Results

A TMA of 175 PDAC specimens was performed to assess gp-130 expression on pancreatic tumors.

### Clinicopathological parameters

The study population consisted of 94 males and 81 females, ranging from 32 to 88 years (median 68.4 years). Most patients were older than 60 years (76%) and underwent either a Whipple procedure (34.9%) or a pylorus-preserving partial pancreatoduodenectomy (44.6%) for tumors in the head of the pancreas. As shown in Table 1, most of the tumor samples showed advanced tumor infiltration (pT3 or pT4 = 84.6%) and lymph node involvement (pN1 = 64%), whereas 8.6% of the patients had already developed distant metastases. The median number of lymph nodes analyzed was 13 (range 0–41). The histopathological examination showed high-grade tumors (G3) in the majority (66.9%) of tissue samples and microscopic residual disease after resection in 42.3% of the tumors. Most patients underwent either perioperative chemotherapy (33.2%) or chemoradiotherapy (45.1%), whereas 21.7% of the patients had no adjuvant therapy. The characteristics of the study subjects are summarized in Table 1.

**Table 1:** Clinicopathological parameters of 175 patients with resected pancreatic ductal adenocarcinoma associated with epithelial and stromal gp130 expression

Characteristics	*n*	gp130 expression* (%)			
	175	Epithelial	*p*	Stromal	*p*
Age					
≤ 60 years	42	80.5	0.765	9.8	1.000
> 60 years	133	78.3		9.3	
Sex					
Male	94	79.3	0.856	9.8	0.857
Female	81	78.2		9.0	
Tumor infiltration					
T1-2	17	87.5	0.527	6.3	1.000
T3-4	158	77.9		9.7	
Lymph node status					
N0	63	78.7	0.974	8.2	0.685
N1	112	78.9		10.1	
Metastasis					
M0	160	78.7	1.000	9.7	1.000
M1	15	80.0		6.7	
Staging					
0-IIa	54	76.9	0.687	9.6	0.952
IIb-IV	121	79.7		9.3	
Grading					
G1-2	58	83.6	0.288	7.3	0.587
G3	117	76.5		10.4	
Residual tumor					
R0	97	71.6	0.015	9.5	0.820
R1	74	87.3		8.5	
Chemotherapy					
No	38	83.8	0.404	8.1	1.000
CTx	137	77.4		9.8	
Radiochemotherapy					
No	96	77.7	0.680	9.6	0.936
RCTx	79	80.3		9.2	

*CTx* chemotherapy, *RCTx* Radiochemotherapy

*gp130 expression includes following immunohistological staining scores: 1 (weak), 2 (moderate) or 3 (strongly positive); Staging according to UICC 2010

### Immunohistochemical analysis

**Fig. 1:**
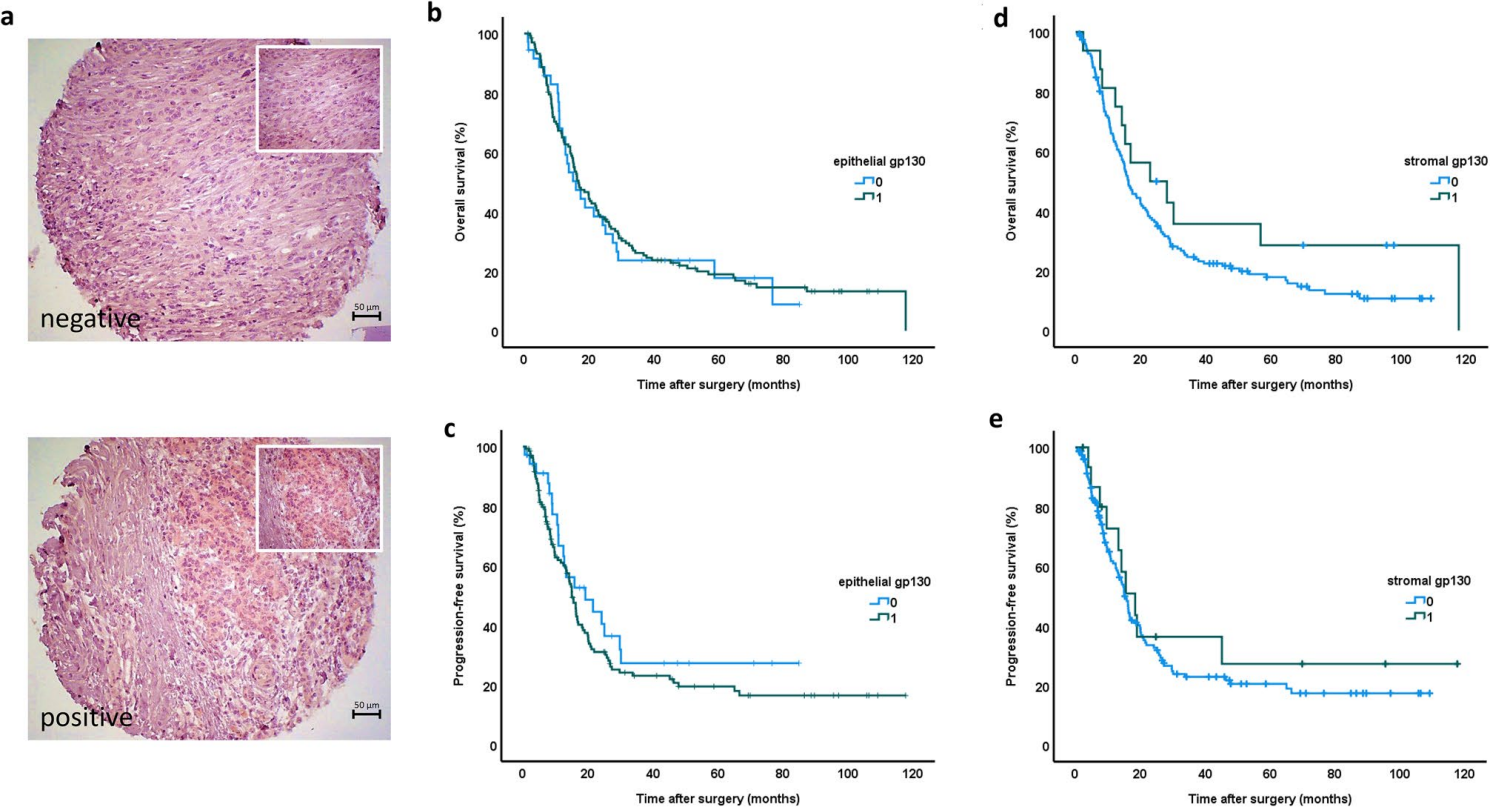
Survival analysis of epithelial and stromal gp130 expression in a Tissue Micro-Array (TMA) from 175 human PDAC samples. Representative immunohistochemical staining showing gp130-positive and -negative TMA tumor samples of patients with PDAC (**a**). Epithelial gp130 expression in human PDAC samples is not associated with overall (**b**) or progress-free survival (**c**). Stromal gp130 expression in human PDAC samples is not associated with overall (**d**) or progress-free survival (**e**); original magnification × 200). Kaplan–Meier curves

Epithelial gp130 expression was found in 78.8% of the tumor samples, and stromal gp130 expression in 9.4% of the tumors. Representative examples of immunohistochemical staining of PDAC tissue micro-arrays for gp130 proteins are shown in Fig. 1a. Epithelial gp130 expression was significantly more frequent in patients with microscopic residual tumor (87.3% with R1 status vs. 71.6% with R0, *p* = 0.015). Apart from residual tumor status (R status), no significant correlation of clinicopathological parameters with the epithelial or stromal gp130 expression was found (Table 1).

### Survival analysis

The median overall survival of patients with PDAC was 16.3 months [interquartile range (IQR) 8.7–37.7], and the mean overall survival was 33.3 months. The median progress-free survival (PFS) was 15.5 months (IQR 8.1–30.1), and the mean progress-free survival was 34.6 months.

### Correlation of epithelial gp130 expression in human PDAC tissue with patient survival

The median overall survival for PDAC patients with no epithelial gp130 expression was 15.9 (IQR 10.7–30.0) months, whereas for patients expressing epithelial gp130 in the pancreatic tumors was 16.7 (IQR 8.5–37.7) months (*p* = 0.830). Patients expressing epithelial gp130 showed a trend for poorer progress-free survival (median 15.0 months, IQR 7.1–29.7) than patients with no epithelial gp130 expression (median 19.1 months, IQR 10.5–31.8) but without significant prognostic relevance (*p* = 0.247). The corresponding survival curves are shown in Fig. 1b and c.

### Correlation of stromal gp130 expression in human PDAC tissue with patient survival

The median overall survival for PDAC patients with no stromal gp130 expression was 16.2 (IQR 8.7–34.2) months, whereas for patients expressing stromal gp130 in the pancreatic tumors was 22.9 (IQR 12.2–117.6) months (*p* = 0.144). While patients with no stromal gp130 expression showed poorer survival than patients with stromal gp130 expression, this difference did not reach significance. The median progress-free survival for patients without and with stromal gp130 expression was 15.0 (IQR 7.7–29.8) and 18.4 (IQR 9.6–33.7) months, respectively (*p* = 0.469). Stromal gp130 expression showed no significant prognostic relevance). The corresponding survival curves are shown in Fig. 1d and e.

### SC144 binds specifically to gp130

**Fig. 2:**
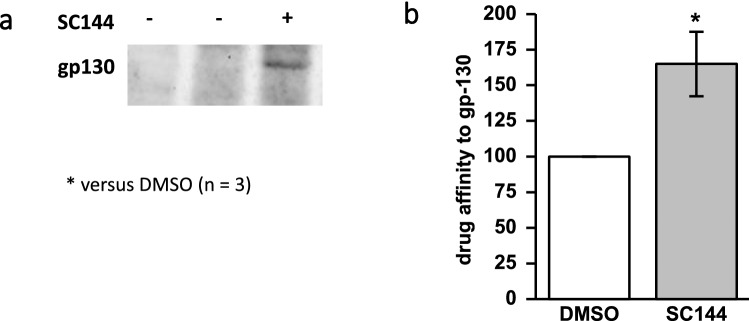
SC144 binds specifically to gp130 in L3.6pl human pancreatic cancer cells. DARTS assay demonstrates the protein–ligand interaction between SC144 and gp130. Binding of SC144 protected gp130 against pronase digestion, which is shown by Western blot (**a**) and densitometric quantification (**b**) (*n* = 3); *p < 0.05; *t* test

DARTS assay was performed on L3.6pl cells to identify and assess the protein–ligand interaction between SC144 and gp130 in vitro. Western blotting (Fig. 2a) and densitometric quantification revealed that the gp130 protein signal was 65 ± 23% more pronounced in SC144-treated samples compared to solvent-treated (DMSO) controls (set 100%) (Fig. 2b). This proves that SC144 binds specifically to gp130 in L3.6pl cell lysates and by this reduces the protease susceptibility of gp130.

### SC144 inhibits pancreatic cancer cell proliferation and viability in a dose-dependent manner

**Fig. 3:**
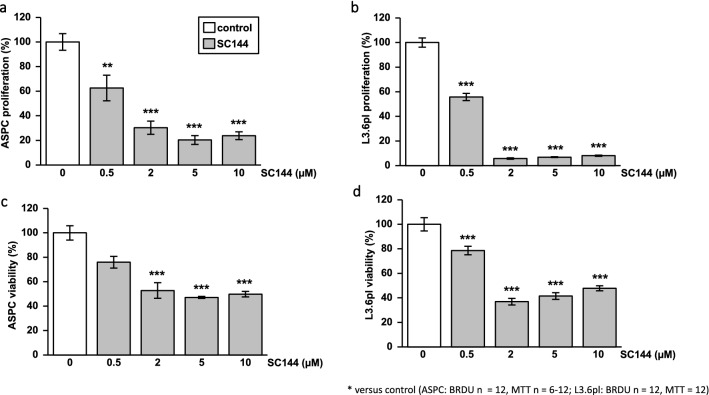
SC144 inhibits L3.6pl and AsPC-1 pancreatic cancer cell proliferation and viability in a dose-dependent manner. BrdU proliferation assays under SC144 treatment in different concentrations in AsPC-1 (**a**) and L3.6pl (**b**) pancreatic cancer cells show proliferation inhibition by SC144 (*n* = 12). MTT cell viability assays under SC144 treatment in different concentrations in AsPC-1 (**c**) and L3.6pl (**d**) cells show reduced viability (*n* = 12); **p < 0.01, ***p < 0.001 versus untreated control; ANOVA with Bonferroni-corrected post hoc tests

Tumor growth depends on the proliferation and viability of cancer cells. Therefore, the SC144 effect on proliferation and viability of AsPC-1 and L3.6pl pancreatic cancer cell lines was assessed in BrdU and MTT assays, respectively. In both AsPC-1 and L3.6pl pancreatic cancer cell lines, SC144 significantly inhibits cell proliferation and viability in a dose-dependent manner. A low dose of 0.5 µM SC144 was already effective in reducing proliferation and viability. In both cell lines, the maximum inhibitory effect was achieved with 2 µM SC144 and could not be enhanced by higher doses of 5 or 10 µM (Fig. 3a–d).

### SC144 suppresses IL-6 and OSM-induced STAT3^Y705^ signaling

**Fig. 4:**
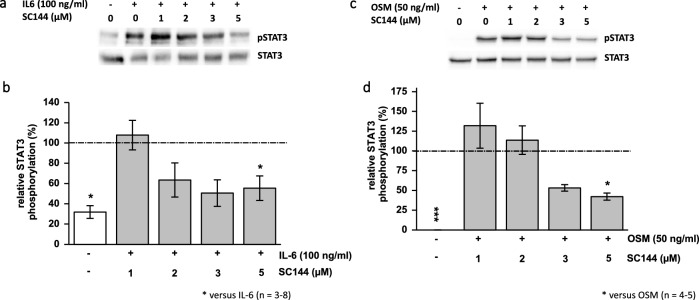
SC144 suppresses IL-6- and OSM-induced STAT3^Y705^ phosphorylation in L3.6pl human pancreatic cancer cells. Representative Western blots (**a**) show that SC144 inhibited IL-6-induced STAT3^Y705^ phosphorylation in L3.6pl cells in a dose-dependent manner. Densitometry revealed that this inhibition was significant after 24 h of treatment (**b**) (*n* = 3–8). SC144 inhibited OSM-induced STAT3^Y705^ phosphorylation in L3.6pl cells in a dose-dependent manner (**c**) which was significant after six hours of treatment (**d**) (*n* = 4–5); *p < 0.05 versus IL-6, **p < 0.01, ***p < 0.001 versus OSM; ANOVA with Bonferroni-corrected post hoc tests

Western blot analyses revealed STAT3^Y705^ phosphorylation was stimulated by 100 ng/ml IL-6 or 50 ng/ml OSM in L3.6pl cells after 24 or 6 h of challenging. We evaluated different SC144 concentrations (1–5 µM) to inhibit the IL-6- or OSM-induced STAT3^Y705^ phosphorylation. Representative Western blots are given in Fig. 4a and c. Treatment with 2 µM SC144 or more showed an inhibitory effect, but only 5 µM SC144 suppressed significantly the IL-6 or OSM-induced activation of STAT3^Y705^ (Fig. 4b and d).

## Discussion

In the present study, we identified gp130 as a novel potential drug target for PDAC therapy. We proved that gp130 is expressed in the epithelium of most human PDAC samples, making it a valid candidate for therapeutically targeting by SC144. Furthermore, we showed that the small molecule gp130 inhibitor SC144 suppresses the PDAC cell viability and proliferation and inhibits IL-6 and OSM-induced gp130/STAT3 signaling in vitro.

Our previous study on tissue micro-arrays from a cohort of 175 patients with PDAC showed that STAT3, phosphorylated STAT3, and IL-6 were expressed in more than half of the examined pancreatic tumors, supporting the importance of this pathway in pancreatic cancer (Pozios et al. 2018). However, the expression of gp130 was not examined in pancreatic tumors before. In the present study, we proved in a cohort of 175 patients that gp130 was expressed in the epithelium of most of the examined pancreatic tumors, which validates the role of gp130 as a promising chemotherapeutic target in patients with PDAC. Our survival analysis demonstrated a poorer prognosis for patients with gp130 expression in tumor epithelium but a trend for a better prognosis for tumors expressing gp130 in tumor stroma. However, these differences did not reach significance in our cohort. A tumor epithelial gp130 expression may indicate an activation of the IL-6/gp130/STAT3 pathway, which acts as a tumorigenic factor in PDAC. In contrast, gp130 stroma expression may contribute to an inflammatory reaction of the tumor microenvironment to inhibit tumor spreading. Epithelial gp130 expression was significantly more frequent in patients with residual tumor (R1 status), indicating aggressive tumor biology, while no other significant correlation of clinicopathological parameters with the gp130 expression was found (Table 1). IL-6/gp130/STAT3 signaling is involved in cancer progression and drug resistance in various human cancers, including ovarian, breast, gastric, and colon (Jones and Jenkins 2018; Xu et al. 2013). In breast cancer, the IL-6/gp130 pathway is frequently activated, promoting breast cancer metastasis and suppressing the anti-tumor immune response (Manore et al. 2022). IL-6/gp130/STAT3 signaling also has an established role in the aggressive and metastatic phenotype of PDAC and constitutes one of the essential signaling cascades in pancreatic cancer initiation and progression (Lesina et al. 2014).

**Fig. 5:**
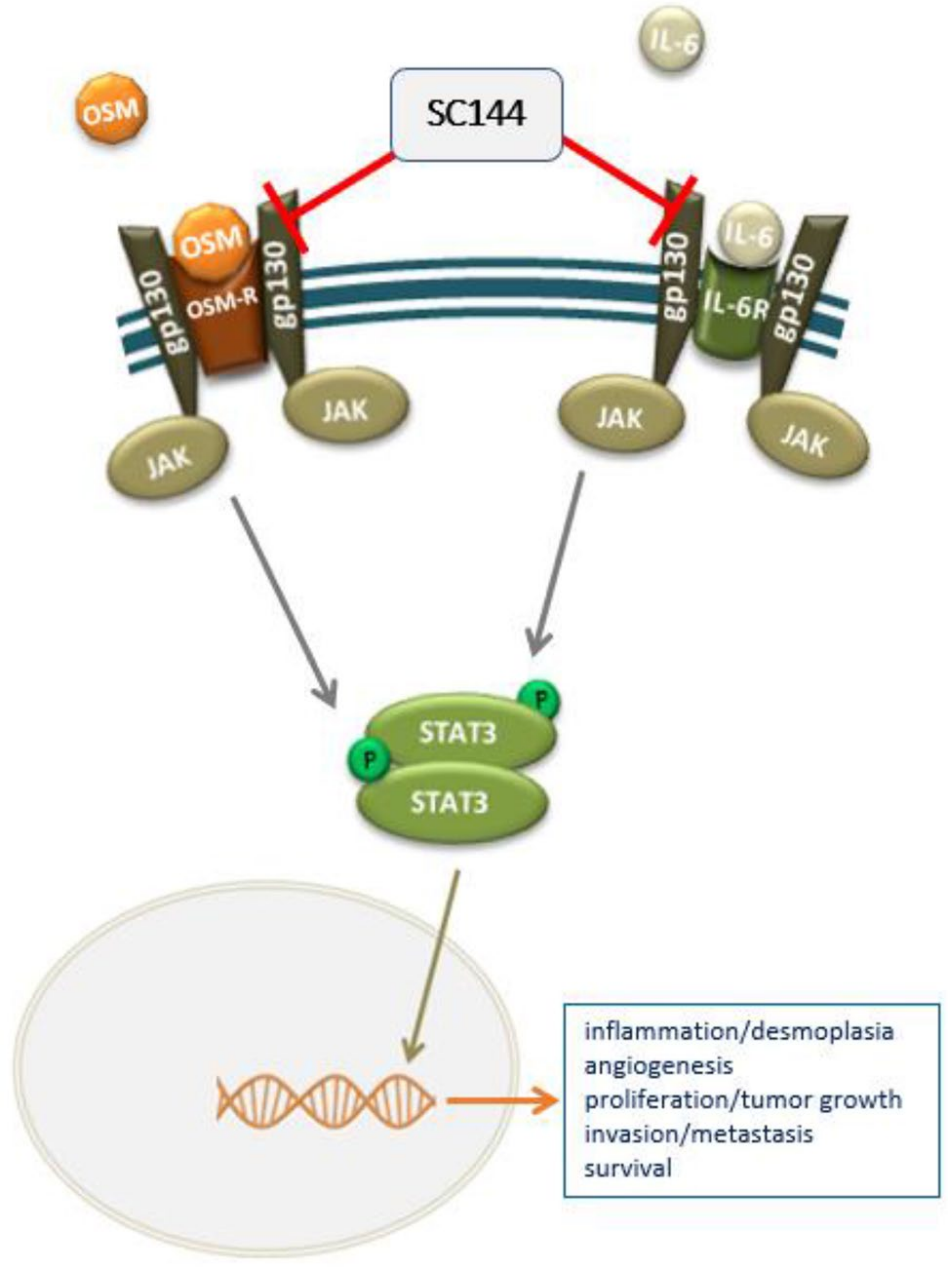
Schematic representation of SC144 mechanism to inhibit IL-6 or OSM/gp130/STAT3 signaling pathway. A working model for the anticancer mechanism of SC144 in pancreatic cancer. SC144 binds gp130, and abrogates gp130 activity, leading to inhibition of IL-6- or OSM-stimulated STAT3^Y705^ phosphorylation and consequently to the suppression of STAT3^Y705^-regulated gene expression

IL-6 mediates part of its functions through the IL-6-receptor complex (IL-6R). The IL-6-receptor is a cell-surface type I cytokine receptor complex consisting of the ligand-binding IL-6R-subunit (chain α) and the signal transducer gp130 (chain β). gp130 (also known as IL6ST or CD130), as a compound of the IL-6R complex, plays a key role in this signaling cascade and, in contrast to the IL-6R-subunit, is ubiquitously expressed. gp130 is the signaling transducer subunit of all IL-6 family cytokines, IL-11, IL-27, IL-31, OSM, cardiotrophin-1, cardiotrophin-like cytokine, ciliary neurotrophic factor, and leukemia inhibitory factor (Heinrich et al. 2003). Gp130 transmits the signal intracellular and activates the tyrosine Janus kinases JAK1, JAK2, and tyrosine kinase TYK2, which leads to the phosphorylation of STAT1 and STAT3. Phosphorylated STAT1 and STAT3 translocate to the cell nucleus (Rincon 2012) and regulate the transcription of target genes involved in proliferation, apoptosis, survival, cell cycle progression, angiogenesis and immunosuppression, playing a pivotal role in many cellular processes (Fig. 5) (Scheller et al. 2006).

SC144 was recently discovered as a novel small molecule gp130 inhibitor with a broad-spectrum anticancer activity (Oshima et al. 2009). Because gp130 is the ubiquitous signaling transducer receptor subunit of all IL-6 family cytokines, SC144 could potentially inhibit STAT3 phosphorylation globally through every gp130-mediated cytokine, achieving a better result on blocking STAT3 effects (Fig. 5).

Within the present study, we demonstrated that SC144 bound specifically to gp130, inhibited proliferation and reduced viability in human PDAC cells. IL-6 and OSM stimulated phosphorylation of STAT3^Y705^. We observed that SC144 suppresses both IL-6 and OSM signaling in a dose-dependent manner in human L3.6pl pancreatic cancer cells. Significant inhibition of IL-6- and OSM-stimulated STAT3^Y705^ phosphorylation was observed by 5 µM SC144. Although statistical significance was only found for 5 µM SC144 at 24 h (IL-6) or 6 h (OSM), also lower doses of 2 and 3 µM SC144 showed a clear trend of STAT3 phosphorylation inhibition respectively. This potential inhibitory effect of SC144 on STAT3^Y705^ signaling, induced by two different cytokines, is promising. However, the role of the other IL-6 family cytokines in pancreatic cancer is unknown and remains to be further analyzed. Previous studies investigated the role of SC144 in other human carcinoma cell lines and showed its cytotoxicity in ovarian, breast, colorectal, and lung adenocarcinoma cell lines (Plasencia et al. 2009). Tumor growth inhibition by SC144 has been reported from xenograft mouse models of breast, colorectal (Plasencia et al. 2009), and ovarian cancer (Xu et al. 2013).

Clinically, in patients with pancreatic cancer, high serum levels of IL-6 correlate with poor prognosis (Lesina et al. 2014), and Oncostatin M (OSM) was overexpressed in the sera of patients with PDAC (Torres et al. 2014). The above data indicate IL-6/gp130/STAT3 pathway as a promising chemotherapeutic target in patients with PDAC. In a previous study of our group, we identified raloxifene to exert an inhibitory effect on IL-6/gp130/STAT3 signaling in PDAC cells and on tumor growth in a xenograft mouse model (Pozios et al. 2020). Recently, various IL-6-directed humanized, murine or chimeric antibodies are under investigation in phase I, II, or III clinical trials as anti-inflammatory or anticancer therapeutic agents, e.g., Olokizumab, Sirukumab, Siltuximab, Clazakizumab, PF-423691, MED15117, and Elsilimomab (Yao et al. 2014). For example, the humanized anti-IL-6R antibody tocilizumab (Actemra, RoActemra in the EU) that has been approved in more than 100 countries for rheumatoid arthritis and other autoinflammatory diseases, is currently examined in combination with gemcitabine/nab-paclitaxel as first-line treatment in patients with locally advanced or metastatic pancreatic cancer (MD 2022). Regarding the fact that gp-130/STAT3 signaling can be activated by numerous different cytokines and growth factors, it can be hypothesized that therapeutically targeting the gp130 subunit might be more effective than blocking IL-6 solely, as other gp130 cytokines may compensate the IL-6 effects.

## Conclusions

In the present study, we proved that gp130 is expressed in the epithelium of most pancreatic cancers, while stromal expression is rare. Moreover, we found that SC144 potently inhibits PDAC progression in vitro and may cause abrogation of IL-6 or OSM/gp130/STAT3 signaling. These results suggest gp130 as a novel drug target for pancreatic cancer therapy.

## Data Availability

The data used to support the findings of this study are available from the corresponding author upon reasonable request.

## Abbreviations

IL-6: Interleukin 6
OSM: Oncostatin M
PDAC: Pancreatic ductal adenocarcinoma
gp130: Glycoprotein 130
STAT3: Signal transducer and activator of transcription 3
IL-6R: IL-6-receptor complex
sIL-6R: Soluble IL-6 receptor
DMSO: Dimethyl sulfoxide
BrdU: 5-Bromo-2′-deoxyuridine (BrdU)
MTT: 3-(4,5-Dimethylthiazol-2-yl)-2,5-diphenyltetrazolium bromide
SDS: Sodium dodecyl sulfate
DARTS: Drug Affinity Responsive Target Stability (DARTS)
IQR: Interquartile range
